# Recent Developments in Deep Learning for Engineering Applications

**DOI:** 10.1155/2018/8141259

**Published:** 2018-05-10

**Authors:** Athanasios Voulodimos, Nikolaos Doulamis, George Bebis, Tania Stathaki

**Affiliations:** ^1^University of West Attica, Athens, Greece; ^2^National Technical University of Athens, Athens, Greece; ^3^University of Nevada, Reno, NV, USA; ^4^Imperial College London, London, UK

Deep learning is among the most rapidly growing fields enabling computational models of multiple processing layers to learn and represent data with multiple levels of abstraction thus implicitly capturing intricate structures of large‐scale data. The recent surge of interest in deep learning methods is mainly due to the abundance of complex data from different sources (visual, medical, social, and sensor) and in a variety of application domains, but also due to the fact that deep learning approaches have been shown to significantly outperform previous state-of-the-art techniques in several research problems.

The aim of this Special Issue is to present new academic research advances and industrial developments of machine learning with emphasis on deep learning for engineering applications. We received twenty-four (24) submissions and each paper was reviewed by at least two experts. Nine (9) papers were accepted for publication in this Special Issue, covering a variety of topics.

In their paper, J. Kim et al. propose a Long Short-Term Memory Recurrent Neural Network (LSTM-RNN) based deep learning framework for energy disaggregation. The conducted experiments on two public datasets (UK-DALE and REDD) indicate that the proposed framework achieves higher performance rates compared to popular Nonintrusive Load Monitoring (NILM) techniques (including Hidden Markov Model based frameworks).

Q. Lan et al. present an optimized GPU implementation of 3D Convolutional Neural Networks and apply it in a video classification scenario. Comparisons of their approach with cuDNN suggest a significant speedup achieved by the former approach.

Focusing on potential users of brain-computer interfaces (BCI), E. Carabez et al. propose a Convolutional Neural Network (CNN) based architecture for identification and classification of brain activity elicited as P300 waves. The CNN models used are given a novel single trial three-dimensional (3D) representation of the electroencephalogram (EEG) data as input, maintaining temporal and spatial information close to the experimental setup. The derived results show increased accuracy for the proposed architecture and 3D input in single trial P300 classification compared to other approaches in the literature.

In another paper of this collection, Y. Feng et al. present a combined deep learning and reinforcement learning framework for entity and relation extraction from unstructured text. The proposed approach combines bidirectional LSTM, tree-LSTM, and Q-learning technique in a two-step decision process and attains higher recall and accuracy rates compared to state-of-the-art methods on publicly available textual datasets for automatic content extraction.

The application of deep learning for disease estimation on plant images is addressed in the paper of G. Wang et al. The authors propose a series of Convolutional Neural Networks fine-tuned via transfer learning techniques, trained to recognize the severity level of disease on plant images, thus forming a framework that yields promising results.

Convolutional Neural Networks are also an integral part of the framework proposed by J. Feng et al. for geographical scene classification. The described model aims at optimizing the feature extractor, so that it can learn more suitable visual vocabularies from geotagging images, resulting in higher recognition rates than other approaches in the literature.

A brief overview of the most important techniques and applications of deep learning in the field of computer vision is provided in the review paper of A. Voulodimos et al. The authors provide a short presentation and comparative analysis of three major deep learning schemes used in computer vision, that is, Convolutional Neural Networks (CNNs), Deep Belief Networks (DBNs), and Deep Boltzmann Machines (DBMs) and Stacked Autoencoders, and their applications on major computer vision problems including object detection, action and activity recognition, face recognition, and human pose estimation.

Another two papers of the Special Issue also present interesting and innovative achievements in machine learning for engineering, though not strictly following a deep learning approach. X. Zhao et al. propose a strategy for achieving high accuracy in synthetic aperture radar (SAR) automatic target recognition (ATR) tasks. Their approach includes novel pose rectification and image normalization to produce images with less variations, followed by feature extraction using wavelet coefficients. A group of discrimination trees are learned and combined into a final classifier in the framework of Real-AdaBoost. The efficacy of the proposed framework is demonstrated through extensive experiments with the public release database MSTAR.

Finally, X. Nie et al. propose the Chaos Quantum-behaved Cat Swarm Optimization (CQCSO) algorithm as an improvement of the Cat Swarm Optimization (CSO) algorithm. The enhanced version addresses one of the problems of CSO, that is, the fact that it is often trapped in local optima in nonlinear optimization problems with a large number of local extreme values. The authors apply their proposed algorithm in a photovoltaic MPPT application scenario.

*Athanasios Voulodimos* *Nikolaos Doulamis* *George Bebis* *Tania Stathaki*

